# Complete Resolution of Cystic Hygroma with Single Session of Intralesional Bleomycin 

**Published:** 2012-07-01

**Authors:** Muhammad Sharif, Isam Elamin Elsiddig, Fadi Atwan

**Affiliations:** Department of Pediatric Surgery, King Fahad Hospital Albaha, KSA.

**Dear Sir**

Intralesional sclerotherapy as a primary modality of management for cystic hygroma is successfully described in literature. It has many benefits over surgical approach; recurrence being the concern of as much as 20% of patients in which apparent complete excision has been performed. Bleomycin is one of sclerosing agents used as intralesional therapy in cystic hygroma. Complete response usually occurs in multiple sessions of sclerotherapy. Rarely, complete resolution occurs with single session of bleomycin sclerotherapy [1-7]. We share our experience of managing a case of cystic hygroma of neck that completely resolved with single session of bleomycin sclerotherapy.

A 5-day-old male neonate presented with swelling in left side of neck since birth. Swelling was painless and multi-cystic. Transillumination of the swelling was positive. Clinical diagnosis was cystic hygroma of the neck. Ultrasound and CT scan were performed to see the extent of the cystic hygroma. He was kept on follow-up for 1 month when he was again admitted. Aspiration with wide bore needle was done under G/A, followed by intralesional bleomycin sclerotherapy in three quadrant (dose 0.3 mg/kg). Post sclerotherapy course was uneventful except initial increase in size and serous discharge from swelling for 15 days. Later on, all symptoms relieved and at 3 months follow-up, the cystic hygroma had completely resolved leaving behind only a small pigmented area (Fig. 1,2).

**Figure:**
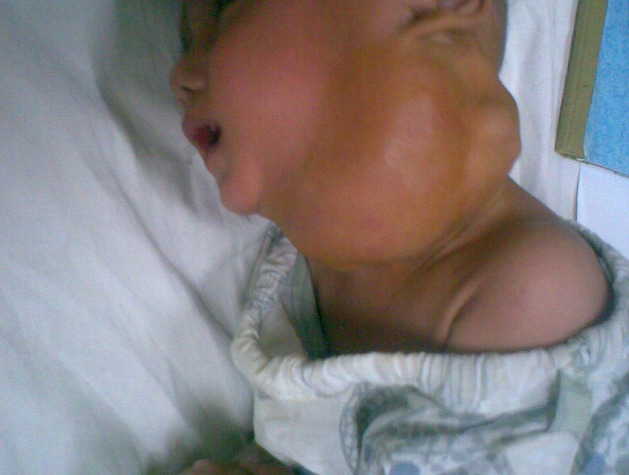
Figure 1: Cystic hygroma before sclerotherapy

**Figure:**
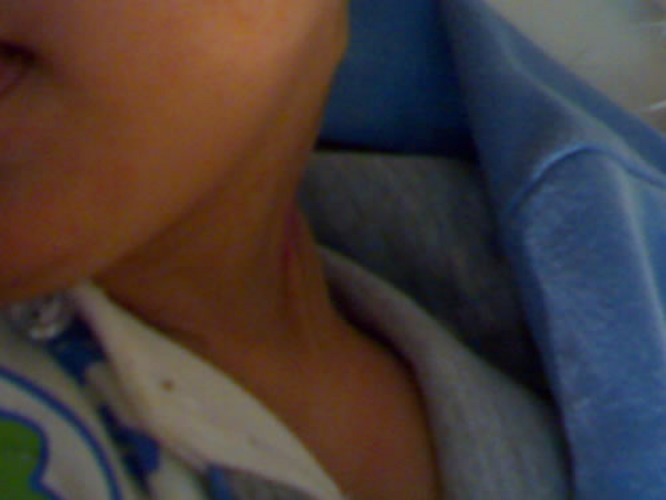
Figure 2: At three months follow-up.

Cystic hygroma is a macrocystic type of lymphatic malformation which is benign and asymptomatic lesion in most of the cases, however, various types of complications can arise in it i.e. recurrent bouts of infection in the lesion, respiratory distress, dysphagia, hemorrhage inside cystic hygroma, sudden increase in the size of lesion, lymph discharging sinus and disfigurement etc. The respiratory distress can be of severe nature necessitating a tracheostomy due to complete or significant laryngeal or tracheal compressions. Intralesional sclerotherapy is an effective treatment modality for the management of cystic hygroma. Surgical excision of the complex cystic hygroma of neck, involving deep structures, is difficult and demanding a meticulous technique and care to avoid per-operative complications. Moreover, recurrence and lymphatic sinus are other problems associated with surgery. Intralesional sclerotherapy with bleomycin or OK-432 is an effective alternate to surgery. With sclerotherapy, complete resolution occurs in about 60% of cases. The sclerotherapy has to be performed multiple times before complete resolution [1-7]. In our case, however, complete resolution occurred with single session of intralesional bleomycin sclerotherapy which is very rare and unusual. We cannot conclude specific reason for complete resolution in our case based on a single case. Proper studies should be designed to identify factors that may help in complete resolution with less sessions of sclerotherapy.

## Footnotes

**Source of Support:** Nil

**Conflict of Interest:** None

